# Imaging coronary plaques using 3D motion-compensated [^18^F]NaF PET/MR

**DOI:** 10.1007/s00259-020-05180-4

**Published:** 2021-01-21

**Authors:** Johannes Mayer, Thomas-Heinrich Wurster, Tobias Schaeffter, Ulf Landmesser, Andreas Morguet, Boris Bigalke, Bernd Hamm, Winfried Brenner, Marcus R. Makowski, Christoph Kolbitsch

**Affiliations:** 1 Physikalisch-Technische Bundesanstalt (PTB), Braunschweig, Berlin, Germany; 2 Klinik für Kardiologie, Charité Campus Benjamin Franklin, Universitätsmedizin Berlin, Berlin, Germany; 3 Berlin Institute of Health, Berlin, Germany; 4 School of Biomedical Imaging Sciences, King’s College London, London, UK; 5 Department of Medical Engineering, Technische Universität Berlin, Berlin, Germany; 6 Department of Radiology, Charité, Universitätsmedizin Berlin, Berlin, Germany; 7 Department of Nuclear Medicine, Charité, Universitätsmedizin Berlin, Berlin, Germany; 8 Department of Radiology, Klinikum Rechts der Isar, Technische Universität München, München, Germany

**Keywords:** Simultaneous PET/MR,, Motion compensation,, [^18^F]NaF cardiac imaging,, Atherosclerosis,, Cardiac and respiratory motion

## Abstract

**Background:**

Cardiac PET has recently found novel applications in coronary atherosclerosis imaging using [^18^F]NaF as a radiotracer, highlighting vulnerable plaques. However, the resulting uptakes are relatively small, and cardiac motion and respiration-induced movement of the heart can impair the reconstructed images due to motion blurring and attenuation correction mismatches. This study aimed to apply an MR-based motion compensation framework to [^18^F]NaF data yielding high-resolution motion-compensated PET and MR images.

**Methods:**

Free-breathing 3-dimensional Dixon MR data were acquired, retrospectively binned into multiple respiratory and cardiac motion states, and split into fat and water fraction using a model-based reconstruction framework. From the dynamic MR reconstructions, both a non-rigid cardiorespiratory motion model and a motion-resolved attenuation map were generated and applied to the PET data to improve image quality. The approach was tested in 10 patients and focal tracer hotspots were evaluated concerning their target-to-background ratio, contrast-to-background ratio, and their diameter.

**Results:**

MR-based motion models were successfully applied to compensate for physiological motion in both PET and MR. Target-to-background ratios of identified plaques improved by 7 ± 7%, contrast-to-background ratios by 26 ± 38%, and the plaque diameter decreased by −22 ± 18%. MR-based dynamic attenuation correction strongly reduced attenuation correction artefacts and was not affected by stent-related signal voids in the underlying MR reconstructions.

**Conclusions:**

The MR-based motion correction framework presented here can improve the target-to-background, contrast-to-background, and width of focal tracer hotspots in the coronary system. The dynamic attenuation correction could effectively mitigate the risk of attenuation correction artefacts in the coronaries at the lung-soft tissue boundary. In combination, this could enable a more reproducible and reliable plaque localisation.

**Supplementary Information:**

The online version contains supplementary material available at 10.1007/s00259-020-05180-4.

## Background

In medical imaging, PET is used in a wide range of different cardiac applications, from the assessment of cardiac viability and perfusion to the recently developed atherosclerotic plaque imaging using [^18^F]NaF [1, 2]. The latter can be used to highlight vulnerable plaques that are likely to rupture and cause myocardial infarction by identifying pathological characteristics such as micro-calcifications and inflammation [3]. Reliable and reproducible quantification of [^18^F]NaF uptake would be a step towards patient- and plaque-specific treatment planning.

Both hybrid modalities PET/CT [1, 4, 5] and PET/MR [6, 7] have been shown to provide a good assessment of coronary [^18^F]NaF uptake. A comprehensive comparison between both hybrid modalities [7] found equally successful plaque identification in aortic valves and coronary arteries. Yet, both hybrid modalities suffer from the main challenge of cardiac PET in clinical applications: the impairing effect of physiological heart motion due to both respiration and cardiac movement. The physiological motion of the heart leads to a PET uptake blurring, which is especially a challenge for coronary plaque imaging due to the small size of the plaques. Besides, motion can lead to a mismatch between attenuation correction (AC) maps and the PET emission data. The proximity of the coronary arteries to the lungs makes this especially a problem for coronary plaque PET imaging. The major difference in attenuation values between cardiac tissue and lung can lead to severe artefacts.

While the straightforward motion compensation approach of gating are very robust, only a small part of the acquired data are used for image reconstruction yielding a low signal-to-noise ratio [1, 8]. Also, it does not address AC misalignment artefacts due to motion [6]. Motion compensation has been proposed to overcome these challenges for a range of other simultaneous PET/MR techniques [9–12]. Improvements have been shown for MR-based ACs using different respiratory positions or allowing for free-breathing [13, 14].

So far, motion compensation techniques in [^18^F]NaF imaging have been PET/CT-based only. The application of motion compensation showed an increase in uptake [15, 16], was extended to successfully improve the test-retest reproducibility of [^18^F]NaF PET plaque imaging [17] and combined with partial volume corrections [18]. However, the sequential PET/CT data acquisition restricts the motion estimation to be performed on motion-resolved low-resolution PET data only, limiting this approach to plaques with high uptake. Simultaneous PET/MR overcomes this challenge by allowing to estimate the motion from high-quality and anatomically detailed images: as of recently, a range of different motion correction schemes is available where motion information is extracted from simultaneously acquired MR data and utilised during PET image reconstruction [19–23]. Yet, so far, there has been no application of cardiorespiratory PET/MR motion correction to [^18^F]NaF imaging.

In this work, we present for the first time MR-based cardiorespiratory motion compensation of simultaneous PET [^18^F]NaF/MR imaging of the coronary arteries. A framework was developed combining advanced 3D MR acquisition with model-based reconstruction techniques and dedicated image registration for motion estimation. From the acquired MR data, patient-specific motion models and dynamic AC maps were generated and applied during PET image reconstruction. This approach minimises motion artefacts in the emission data and ensures accurate alignment between PET and AC data. The framework yields 3D high-resolution and motion-compensated images for both PET and MR. The proposed methods were demonstrated in 10 patients. The effect of motion-corrected image reconstruction (MCIR) on both MR and PET image quality was assessed in uptake-positive plaques by comparing their target-to-background ratio (TBR), their contrast-to-background ratio (CBR) and the tracer visualisation.

## Methods

### PET-MR data acquisition

PET-MR data were acquired as part of the study “Molecular PET/MR–Imaging for detection and characterisation of vulnerable atherosclerotic plaques in coronary arteries” (EA4/052017) approved by the Charité ethics committee. It was performed in accordance with the Declaration of Helsinki. Before taking part in the study, all patients provided written informed consent. The patient cohort consisted of 10 subjects (8 male), mean age 70 ± 7 years, suffering from known or probable coronary artery disease, who were scheduled to undergo cardiac catheterisation. Additional eligibility criteria included MR contrast agent tolerance and age larger than 50 years due to radiation protection.

Data were acquired on a Siemens Biograph mMR hybrid PET/MR scanner. A dose of 169 ± 14 MBq [^18^F]NaF tracer was administered intravenously 104 ± 26 minutes before starting the PET acquisition. The minimum duration of PET acquisition was 30 minutes and was continued until the last MR examination had ended. The average data acquisition time was 45 ± 20 minutes due to patient-specific scan time variations (e.g. because of double-gated 3D MR angiographies). Before the PET scan, an MR-AC scan provided by the vendor was carried out during breath-hold (FOV = 598 × 330 × 271 mm^3^, dx × dy × dz = 2.086 × 2.6 × 2.086 mm^3^, TA = 10.6 s).

The MR data were acquired for 12:25 minutes simultaneously with the PET data and used a T1-weighted, Dixon sequence (TR = 7.57 ms, TE = 2.62/4.13/5.64 ms, FA = 15°) with a double-oversampled 3D radial phase encoding k-space sampling trajectory [24] with a field of view (FOV) covering the entire thorax (FOV = 288 × 288 × 288 mm^3^) at 1.5-mm isotropic resolution. ECG and respiratory signals were acquired simultaneously with the physiological monitoring unit. A T_1_ contrast agent (Gadovist) bolus between 14 and 18 ml was administered before the MR exam. The contrast agent was used to increase the contrast between the blood pool and myocardium and improve the visualisation of the coronary arteries [25].

### PET/MR reconstruction workflow

**Fig. 1:**
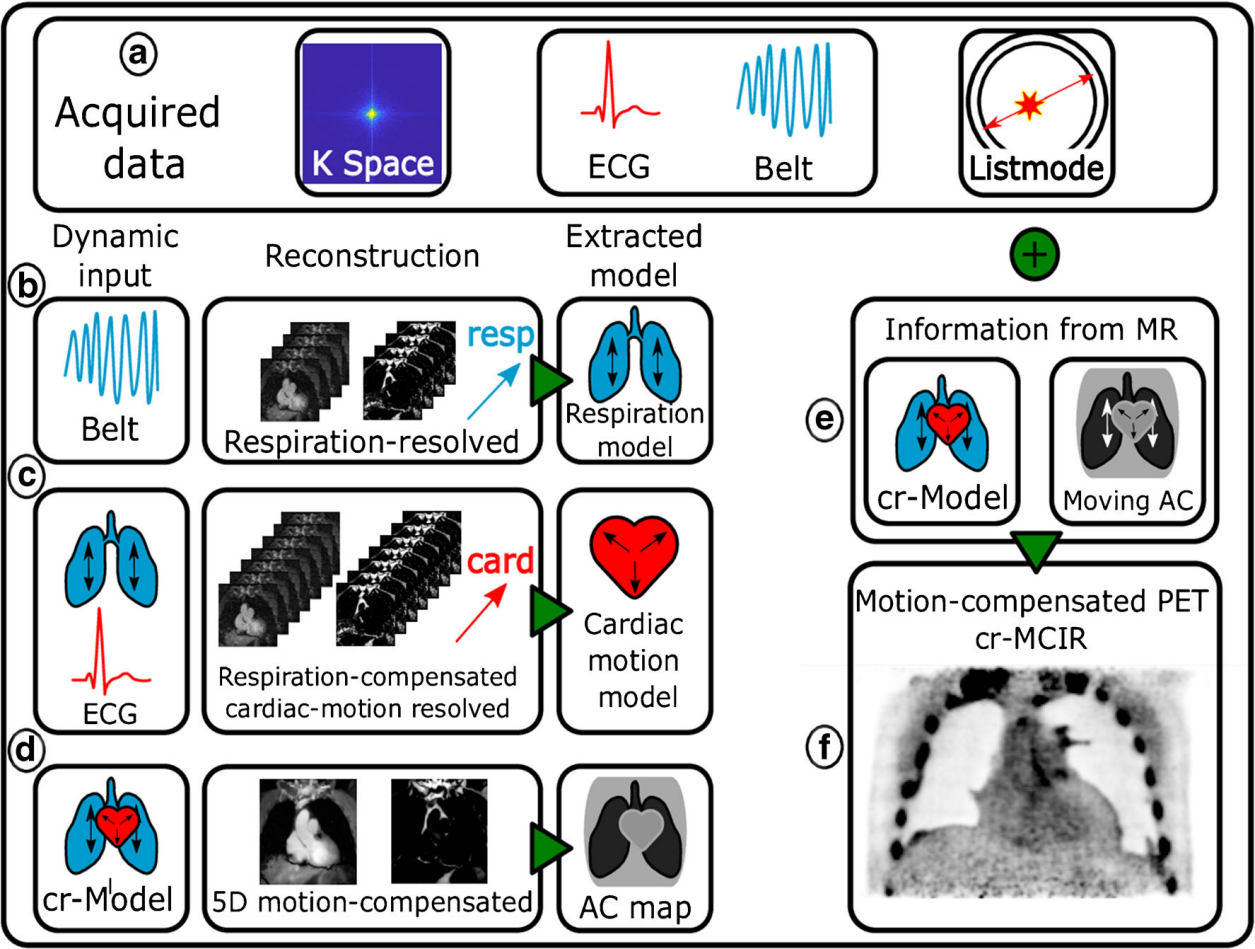
Overview of the reconstruction workflow. The acquired PET/MR data consist of listmode and k-space data, as well as the respiratory belt and ECG as surrogate signals for physiological motion. Three MR reconstructions (**b**–**d**) are performed from which motion information and an attenuation map are extracted. This information is incorporated into the PET reconstruction (**e**) compensating both emission data and attenuation map for motion yielding a cardiorespiratory motion-compensated (cr-MCIR) PET reconstruction (**f**). The cr-MCIR MR (**d**) and PET (**f**) reconstructions are hence in the same motion state and the MR anatomical image can be used to identify the anatomical location of the uptake

An overview of the reconstruction workflow is depicted in Fig. 1. The acquired data (A) consist of MR k-space, PET listmode data and respiratory belt and ECG signal as motion surrogates.

In the first step, motion-resolved MR images are reconstructed from which non-rigid respiratory and cardiac motion models are extracted, respectively. In a second step, the motion models are combined to reconstruct a fully cardiorespiratory motion-corrected image. From this image data, a 4-tissue AC map is generated. Finally, the non-rigid motion cardiorespiratory models are applied in a motion-corrected PET image reconstruction.

The workflow starts by using the belt signal to bin the k-space data into six different respiratory states (B), from which motion-resolved fat and water images are reconstructed. Based on these images, a respiratory motion model is generated using image registration (as discussed in detail in the following). The respiration-resolved reconstructions underlying the respiratory motion model still contain cardiac motion artefacts. Nevertheless, these are similar in all respiratory motion states and hence do not interfere with an accurate estimation of respiratory motion.

Subsequently, data are binned into 12 different cardiac states (C) using the ECG signal and cardiac motion-resolved reconstructions corrected for respiratory motion are performed, using the previously estimated model for respiration. Hence, each reconstructed cardiac state is free of respiratory and cardiac motion artefacts. A model for the cardiac motion is extracted from this image series using the same registration algorithm as for respiration. Both motion models are combined in a third reconstruction (D) correcting for both motion types. Based on this motion-free 3D MR image, a 4-tissue AC map is computed.

Finally, both motion models are transformed from the MR coordinate system to the PET coordinates. Furthermore, the models are extrapolated from the 12-minute window of MR data acquisition onto the whole duration of the PET scan. A motion-corrected PET reconstruction is performed, where the cardiorespiratory motion model is applied to both the MR-based AC map [9] (E) and the emission data, yielding a cardiorespiratory motion-compensated (cr-MCIR) PET image (F).

### PET/MR image reconstruction

The MR data were reconstructed into fat and water content with an iterative model-based reconstruction [26] framework incorporating the effect of chemical shift and using parallel imaging [27], compressed sensing [28], as well as motion information [29]. Motion-resolved images were reconstructed using the respiratory belt or ECG signal as a surrogate to bin data prior to reconstruction based on respiratory amplitude and cardiac phase. Software for MR reconstruction was implemented in MATLAB (The MathWorks, Natick, MA) and Python.

PET image reconstruction was performed with STIR (Software for Tomographic Image Reconstruction). A FOV of 718 × 718 × 258 mm^3^ was reconstructed at a resolution of 2.09 × 2.09 × 2.03 mm^3^ using a motion-corrected, iterative 3D ordered subset expectation maximisation algorithm with 21 subsets and 3 full iterations, and a 4-mm isotropic 3D Gaussian post-filtering [30]. PET MCIR reconstructs one motion-free image from listmode data acquired in different motion states. During each iteration, the current image estimate is non-rigidly transformed into the individual motion states using the cardiorespiratory motion models extracted from MR. Subsequently, its sinogram is computed, using an equally motion-transformed AC map, and compared to the acquired PET sinograms. This approach yields a cardiorespiratory motion-corrected image that is consistent with acquired PET emission data and mitigates AC misalignment artefacts. PET scatter radiation estimates were performed using SIRF [31] (Synergistic Image Reconstruction Framework).

### MR-based motion models and attenuation correction

Respiratory and cardiac motion models were generated using motion-resolved reconstructed MR images. A dedicated image registration algorithm [21, 32] was used to generate a non-rigid motion model based on both the MR fat and water modes. Both water and fat image series enter the registration in the underlying optimisation problem’s cost function:

$$ C\left({m}_t\right)=w\ast S\left({I}_W\left({m}_t(x),t\right),{I}_W\left(x,0\right)\right)+\left(1-w\right)\ast S\left({I}_F\left({m}_t(x),t\right),{I}_F\left(x,0\right)\right)+r\ast R\left({m}_t\right) $$

where S(.) is a similarity metric (in this case normalised mutual information), I_W/F_ (x,t) is the water and fat image in motion state *t*, m_t_(x) is the coordinate transformation relating the motion state *t* with the reference motion state *t* = 0 and R(m_t_) is a regularisation term and w and r weights associated with the different terms. The rationale behind using both image modes for the registration is that while the water image describes the motion of coronary arteries and heart muscle, the surrounding fat tissue provides complementary information with very high contrast. Additionally, the undersampling artefacts due to motion binning are different in both images such that using both image content acts as additional regularisation. For this study, *w* = 0.5 was used, weighting water and fat images the same. The motion model was generated by registering the individual motion states to the reference phase. Both motion types were combined into a cardiorespiratory motion model concatenating the two individual transformations. Cardiac motion was corrected to yield images in end-diastole, while respiration was corrected to either end-exhale or end-inhale depending on which state was more prevalent in the surrogate signal.

PET data were acquired for longer than MR data. To extend the obtained motion models onto the entire duration of the PET scan, the respiratory belt was used as a respiratory motion surrogate. Nevertheless, an MR self-navigator was available and used to improve the correlation [22] of the belt signal with the respiratory motion of the heart: in the time window during the MR acquisition, a time shift for the belt signal maximising the cross-correlation between belt and self-navigator is determined. This shift is then applied globally to the belt signal. In each case, the computed shift was much smaller than any period of respiration encountered during the exams.

The MR-based 4-tissue AC map was extracted from the MR cr-MCIR image using a custom segmentation algorithm. A k-means clustering algorithm [33] was used to segment fat, soft tissue, lung tissue and air. MR signal voids caused by stents were automatically inpainted using morphological operations. As the arms were not covered in the MR FOV, these were inserted from the vendor AC map. This process is displayed in the supplementary Fig. S1.

The custom AC map was used for both reconstructing motion-averaged (AVG), i.e. without the application of any motion model during reconstruction and cardiac and respiratory motion-compensated PET images (cr-MCIR). Using the MR cr-MCIR images to calculate the AC map for both AVG PET and cr-MCIR PET ensures that differences in image quality between these two reconstructions are predominantly due to physiological motion artefacts in the emission data.

### PET/MR image quality assessment

The quality of MR images for both water and fat fraction was assessed visually between AVG, respiratory motion-compensated, i.e. only the respiratory motion model was applied during reconstruction (r-MCIR), and cr-MCIR reconstructions. An increase in coronary vessel sharpness and the position of the diaphragm was used to determine a successful generation of the dual motion model.

All PET reconstructions were converted into SUV. The STIR open-source reconstructions were compared qualitatively to reconstructions provided by the Biograph mMR in terms of uptake position, noise level and artefacts generated by AC misalignment.

The effect of motion compensation was evaluated quantitatively for all detected plaques based on their TBR, CBR and diameter (D). Uptake-positive plaques were detected visually by an experienced observer. Plaques were identified as clearly visible, coherent tracer uptake structures following the coronary vessel tree. An independent method to confirm that the uptake was a plaque was not available. Both 2D axial and coronal slice through the plaque centre were selected from both AVG and cr-MCIR images. Subsequently, the same plaque in all images was marked with a rectangular ROI. The plaque signal *s* was computed in a 5 × 5 pixel patch around the maximum SUV value in the ROI. To exclude background pixels from the patch, an automated threshold value inside the patch was computed using Otsu’s method [34]. The background signal *b* was extracted from an independent coronary slice and measured as the blood signal average in an ROI in the left ventricle: *b* = 〈*SUV*〉_*ROI left ventricle*_. The TBR was calculated as $TBR=\frac{s}{b}$ and CBR as $CBR=\frac{\left|\mathrm{s}-\mathrm{b}\right|}{\upsigma \left(\mathrm{b}\right)},$ where *σ*(*b*) is the standard deviation of the background. TBR and CBR values generated from both the axial and coronal view of the plaque were averaged to include the effect of correcting motion both in and through transversal planes.

To determine the diameter *D*, a line profile *l*(*x*) perpendicular to the coronary artery was extracted from the coronary slices and fitted with a model containing a Gaussian for the plaque and a linear term and a constant locally describing the scatter background: $l(x)=a\cdotp {e}^{-\frac{{\left(x-b\right)}^2}{c^2}}+d\cdotp x+e$. *D* was determined as the full-width at half maximum of the fitted Gaussian curve: $D=2\sqrt{2\mathit{\log}2}\cdotp c.$ The parameters were constrained to be positive and *d* ∈ [−1, 1].

Statistical analyses between AVG and cr-MCIR data were performed using Python. To test the null hypothesis that the values of both AVG and cr-MCIR originate from the same underlying distribution for the case of normal data, a Wilcoxon signed-rank test was performed. *p* values smaller than 0.05 were considered statistically significant.

## Results

### MR reconstruction and motion model generation

**Fig. 2:**
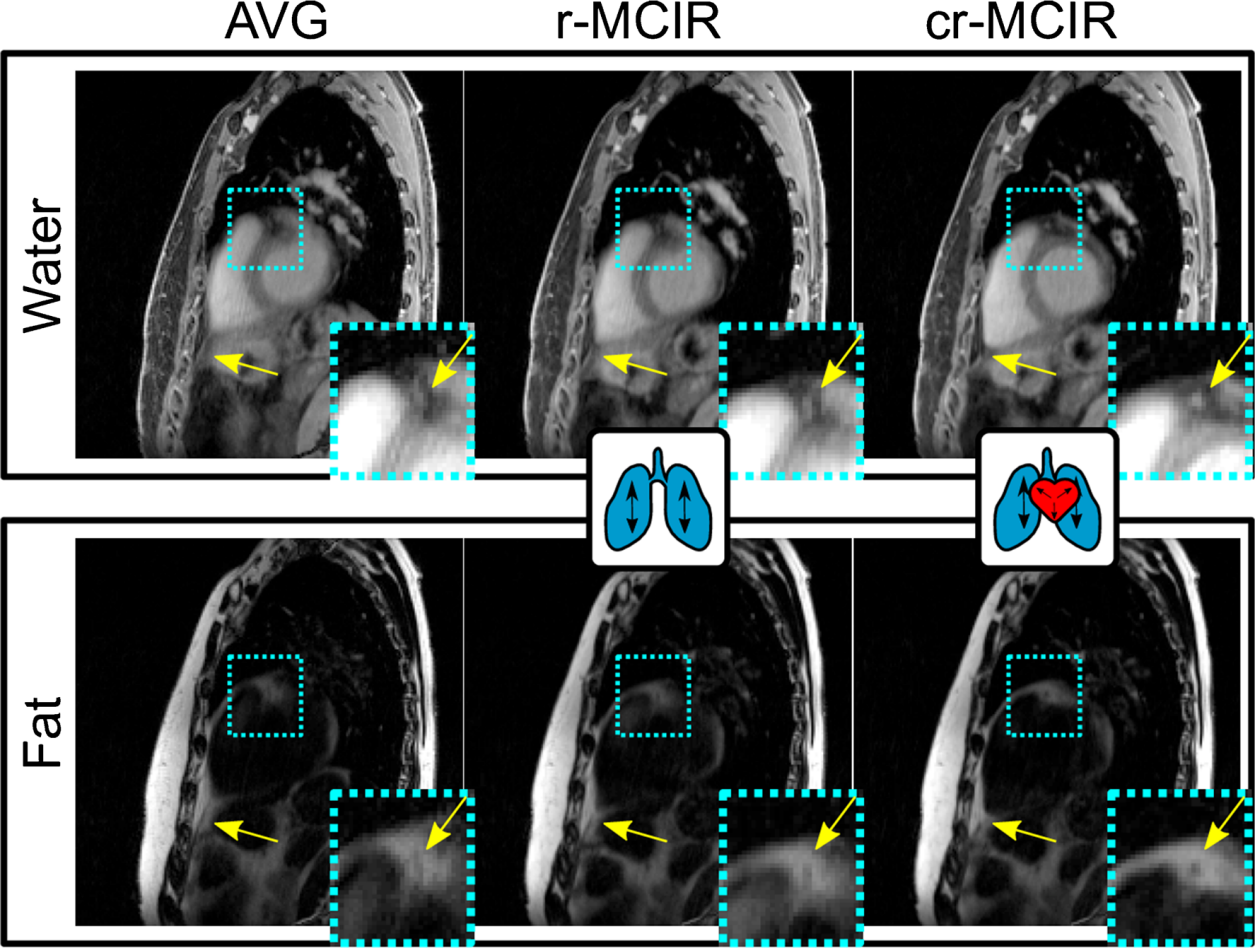
MR images in sagittal view of one exemplary dataset with reconstructed water (top) and fat (bottom) content. The motion-averaged reconstruction (AVG, left) is compared to respiratory (r-MCIR, centre) and cardiorespiratory (cr-MCIR, right) reconstruction. Cyan boxes indicate enlarged areas depicted in the lower right corner reconstruction. Image quality increases with the inclusion of more motion information from AVG over r-MCIR to cr-MCIR for both the water and fat images especially in the coronary arteries and apex (yellow arrows)

The results of the MR motion correction are displayed in Fig. 2. R-MCIR improved the visualisation of the coronary vessel, which can be seen especially for the fat image (small insert in Fig. 2). Nevertheless, there is still residual motion blurring which was further reduced using cr-MCIR.

### PET motion compensation

**Fig. 3:**
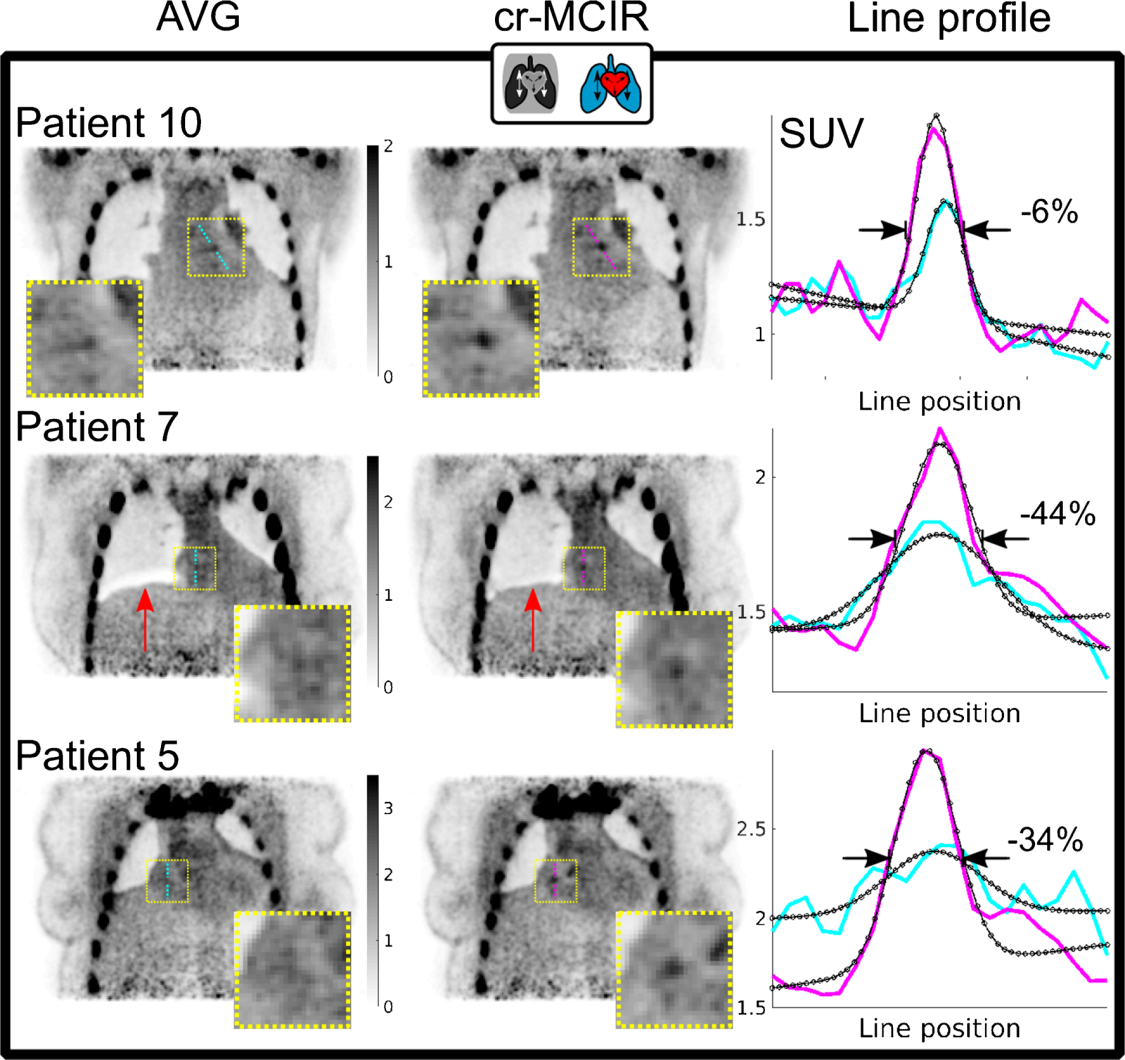
Comparison between PET AVG (left column) and cr-MCIR (centre column) for patients 10, 7 and 5. Cyan and magenta lines indicate the position where line profiles (right column) were extracted. The fit is overlayed in black. Compared to AVG, cr-MCIR leads to an increase in the maximum uptake value and a decrease of plaque width *D*. The decrease ranged between −6 and −44% for the displayed cases. Patient 7 shows an artefact due to misalignment between AC map and PET emission data for AVG which is corrected by using cr-MCIR (red arrow)

The effect of cr-MCIR in PET for three patients is depicted in Fig. 3. The AVG and cr-MCIR reconstructions are compared for three slices showing coronary uptake. Line profiles in cyan for AVG and magenta for cr-MCIR are plotted and an increase in SUV_max_ is visible in the line profiles. The line profile fit based on which *D* is calculated is indicated as a black curve, and for the three presented cases, *D* was reduced by 6, 44 and 34% respectively. Furthermore, one can see that for patients 10 and 5, there was already a very good match between the emission data and the AC map as very little imprint artefacts are visible. The AVG reconstruction for patient 7, however, has an artefact indicated by a red arrow due to the misalignment between PET emission data and AC map that was strongly reduced by cr-MCIR.

**Fig. 4:**
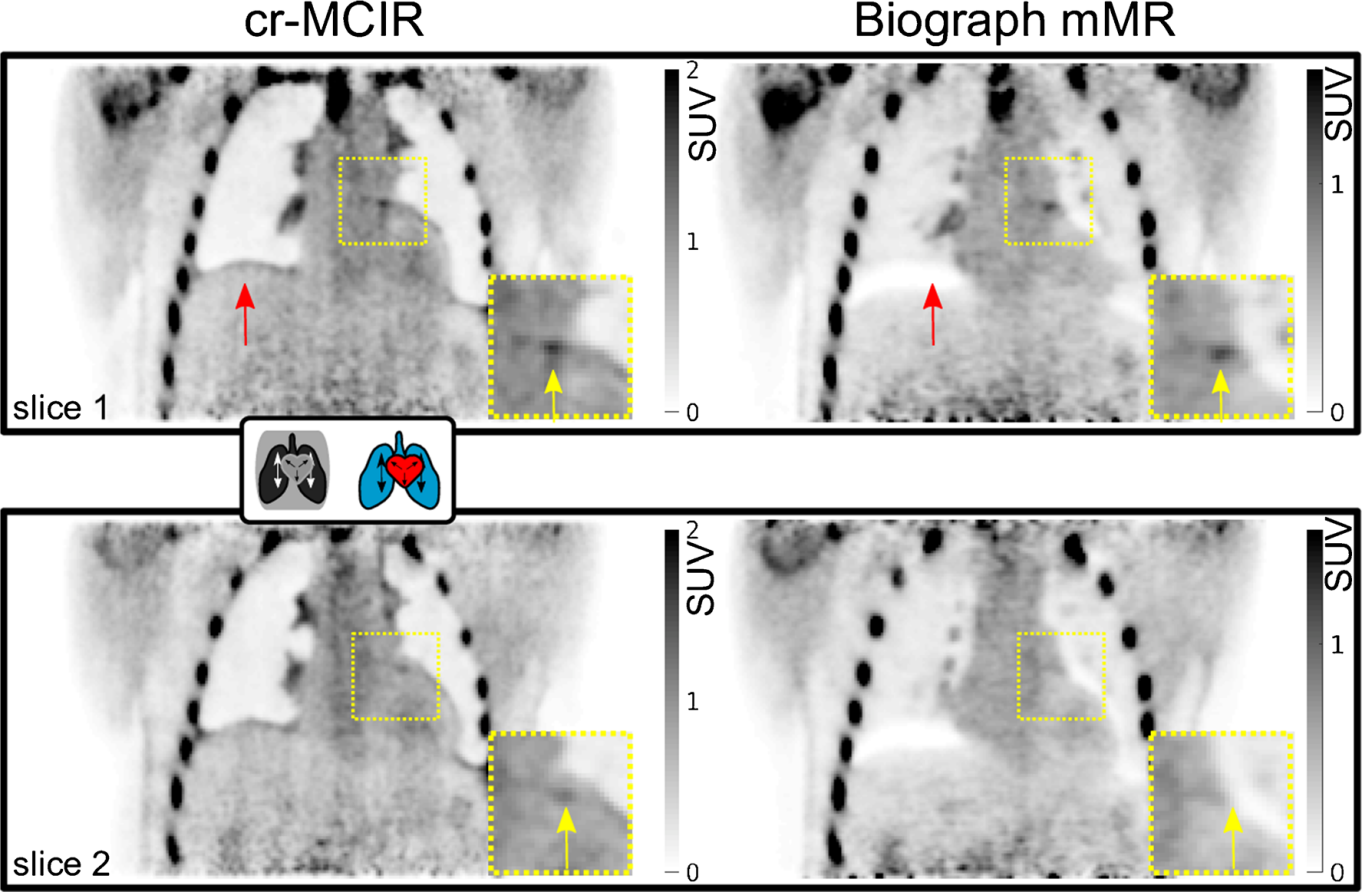
Result of the PET cr-MCIR application to patient 1. An enlarged ROI is marked by the dashed yellow square. Top, coronal slice 1; bottom, coronal slice 2. Left, cr-MCIR. Right, Biograph mMR vendor reconstruction. Uptake in the left coronary artery is highlighted by yellow arrows, and red arrows indicate artefacts due to an AC mismatch. The cr-MCIR shows reduced AC mismatch compared to the Biograph mMR images. For the vendor reconstruction, the strong AC data mismatch due to physiological motion means the uptake in the coronary plaque is not visible anymore in the second slice (bottom row). Scanner and STIR reconstructions required a different window setting for comparable contrast in the coronary uptake

**Fig. 5:**
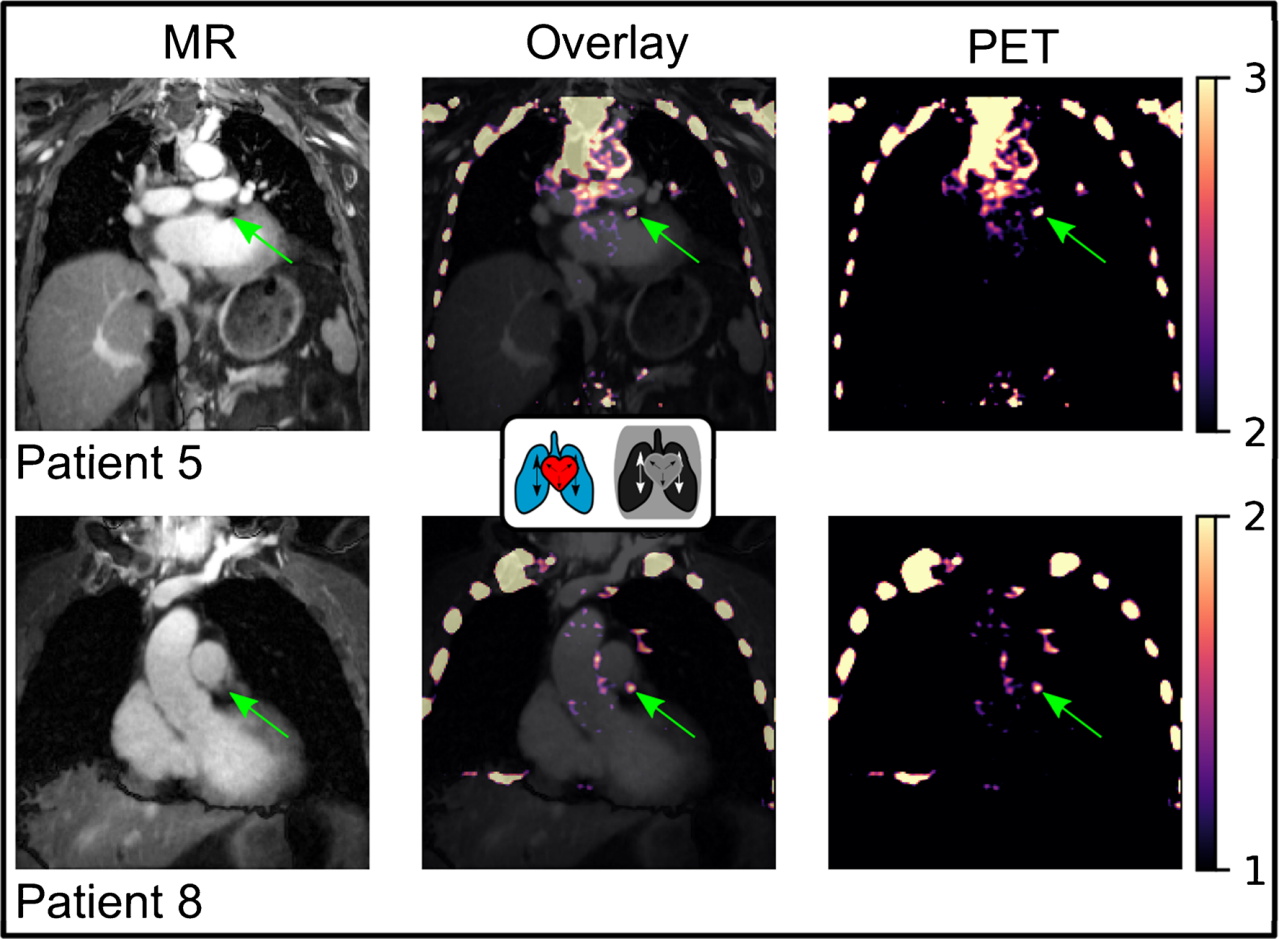
PET/MR overlay for patients 5 and 8 with uptake in stents (green arrows). All images are cr-MCIR. PET images were reformatted to the MR coordinates. Left column, MR water images. Central column, overlay of both modalities. Right column, PET images only. The colorbar corresponds to the PET window in both images. In this case, the anatomic position of the uptake is obscured by the signal void generated by the stent. However, the automated inpainting of the stent voids during AC map generation mitigates potential AC artefacts with respect to tracer uptake within stents

A comparison between the AVG, cr-MCIR and Biograph mMR images is depicted in Fig. 4. Two different coronal positions are displayed, both of which contain uptake in the left coronary artery. AVG and cr-MCIR reconstructions achieved a comparable quality of the PET uptake of the peripheric thorax region, visible when regarding uptake in the ribcage and shoulders. The overall background noise due to scatter radiation was lower in the Biograph reconstructions such that slightly different window settings were required to create the same visibility of the plaque uptake. The misalignment between the AC map and emission data is especially visible in the scanner reconstruction at the right hemidiaphragm (red arrow, “banana artefact”). Those impaired the visualisation of the coronary uptake which was located right at the edge of the heart and thereby obscured the actual extent of the uptake along the vessel indicated by the yellow arrows. An overlay of MR and PET is depicted in Fig. 5. For patients 5 and 8, a coronal slice is depicted where the PET image was reformatted to the MR coordinate system. Both patients showed uptake in a stent that caused an artefact in the MR image. Despite this void, a reliable MR-based AC map could be estimated allowing for accurate PET quantification and visualisation of plaque uptake. However, the exact anatomic position could not be determined in the MR image. Especially for patient 8, the signal void was much larger than the uptake.

### Quantitative PET image assessment

In the 10 patient datasets, 10 coronary plaques were identified in 9 subjects. A comprehensive overview of the computed image quality metrics is given in Table 1. The measured TBR in the 10 plaques ranged between 1.11 and 3.1 for AVG and 1.21 and 3.41 for MCIR reconstruction. The average increase in TBR was +7 ± 7% using cr-MCIR. The Wilcoxon signed-rank test yielded a *p* value of *p* < 0.03.

**Table 1:** Overview of quantitative data

Patient	TBR_stat_	TBR_dual_	TBR_ratio_	CBR_stat_	CBR_dual_	CBR_ratio_	FWHM_stat_	FWHM_dual_	FWHM_ratio_
1	1.34	1.51	13	2.59	4.24	64	12.2	11.63	−5
2	1.45	1.51	4	4.43	4.80	8	12.54	9.9	−21
3	1.30	1.21	−7	3.22	2.30	−29	7.6	7.84	3
4	3.10	3.41	10	15.15	18.76	24	23.71	17.91	−24
5	1.11	1.24	12	1.02	2.12	107	18.72	12.39	−34
5	1.58	1.68	6	5.46	5.93	9	12.57	5.47	−56
6	2.54	2.50	−2	9.28	8.80	−5	11.87	9.91	−16
7	1.37	1.48	8	4.49	5.72	27	23.39	13.09	−44
8	1.35	1.47	9	4.43	5.80	31	16.43	12.62	−23
10	1.54	1.76	14	5.06	6.13	21	10.07	9.43	−6
Mean	**1.67**	**1.78**	**6.70**	**5.51**	**6.46**	**25.70**	14.91	11.02	−22.60
Std.	0.63	0.68	6.75	4.01	4.74	37.50	5.48	3.37	18.23
			***p*** **= 0.02**			***p*** **= 0.04**			***p*** **= 0.01**

Patient numbering is the same as in the figures

The CBR ranged between 1.02 and 15.15 for AVG and 2.12 and 18.76 for cr-MCIR reconstructions with a mean increase of +26 ± 38% for cr-MCIR compared to AVG. The Wilcoxon signed-rank test applied to the data yielded a *p* value of *p* < 0.05.

D ranged between 7.6 and 23.4 mm for the AVG and 5.47 and 17.91 mm for the cr-MCIR reconstructions. On average, cr-MCIR decreased D by 23 ± 18%.

The Wilcoxon signed-rank test yielded a *p* value of *p* < 0.02.

## Discussion

In this work, we presented an MR-based motion compensation framework and its application to coronary [^18^F]NaF PET/MR imaging. The data-driven patient-specific cardiorespiratory motion models enabled MCIR for both modalities which improved the assessed image quality metrics TBR, CBR and plaque width significantly.

The quantitative assessment yielded an average increase in TBR of 7 ± 7% and CBR of 26 ± 38%. The plaque localisation in cr-MCIR reconstructions was improved compared to AVG reconstructions, reducing the diameter *D* on average by 23 ± 18%. Statistical tests on all these quantities yielded significant differences between AVG and cr-MCIR. The estimated motion model also improved the anatomical visualisation of coronary arteries in the MR cr-MCIR where both cardiac and respiratory motion contributed to an increase in the visibility and sharpness of the coronary vessels and fat surrounding the heart. The improvement of TBR and CBR depends on the location of the plaque, the motion amplitudes and motion cycles which strongly vary between patients.

Furthermore, it could be shown that a dedicated motion modelling of the AC was able to minimise any mismatches between the AC map and emission data, allowing for accurate visualisation of plaque uptake in the PET images. A first improvement, as shown in Fig. 3, can be achieved by obtaining the AC map from a cr-MCIR MR image that is motion-corrected to the most prevalent respiratory state. Further improvement is achieved by adapting the AC map to the different motion states as part of PET MCIR (patient 7 in Fig. 3). In contrast to that, the AC map for vendor reconstruction was acquired during a breath-hold. Figure 4 depicts an example where the breath-hold position was very different from the free-breathing position of the heart during PET data acquisition. This led to a strong AC mismatch severely impairing the visualisation of the uptake in the plaque, because AC values of the lung were used to correct PET emission data of the heart.

One limitation of the presented work is that the tracer uptake could not be independently identified as coronary plaques. Furthermore, a comparison between the images achieved using motion compensation and cardiorespiratory double-gated reconstructions were omitted. The observed TBRs using the whole dataset were too small to expect any visible plaque uptake in double-gated images. Similar to many in vivo motion-corrected image reconstruction approaches, there was no available ground-truth information on the patient motion available. The estimated motion models were visually inspected to ensure that the computed transformations were physiologically realistic, as well as successful in capturing the motion. While the respiratory motion model is patient-specific, the necessity to extrapolate the respiratory motion model from the time window of MR data acquisition onto the whole PET acquisition potentially reduces its accuracy. Large differences in respiratory amplitudes between the time during which the motion model was generated and the rest of the PET exam could make the application of the motion model less effective [35]. To ensure the validity of the respiratory extrapolation, further analysis was performed. It has been shown that the breathing pattern can be inferred from the distribution of the displacement of the diaphragm [36]. The distributions of the respiratory belt signal were compared in and outside the 12-minute window of MR data acquisition. We did not see a large intra-patient variability between both distributions for any of the 10 patients, suggesting that the extrapolated respiration model is valid. This is depicted for three exemplary patients in supplementary Fig. S3.

This required to use the respiration belt for data binning which generally is less accurate in describing motion states compared to an MR-based self-navigator [35]. Using additional motion surrogates [37] might further improve the application of the motion model to the PET data.

Also, this study neglected point spread function modelling of the PET system. As the scatter radiation used by the vendor system is unavailable, a standard technique [38] was used for its computation. As displayed in Fig. 4, comparable overall image quality was achieved with a slightly higher noise level in our reconstructions than in the vendor images. Being able to use the scatter estimation of the vendor could further improve image quality [39].

Furthermore, as depicted in Fig. 5 and shown by previous studies [7], the MR signal voids caused by stents do prevent the use of the MR as anatomic information to precisely locate uptake. Potential errors, however, that propagate into the MR-based AC map could be efficiently and automatically dealt with by morphological transformations and inpainting. The AC map outside the MR FOV was completed by the vendor-provided attenuation information of the arms. These data, if not available, could also be acquired time-efficiently in a short, free-breathing, extra scan of a few seconds before or after finalisation of the PET exam.

## Conclusions

We presented the development and application of an MR-based motion compensation framework dedicated to [^18^F]NaF PET/MR imaging, yielding cardiorespiratory motion-compensated high-resolution 3D MR and PET images. The presented increase in TBR and CBR, the reduction in plaque width and mitigation of attenuation correction mismatch artefacts could enable a more reproducible and reliable plaque localisation.

## Supplementary Information

ESM 1 (PNG 3881 kb)

High resolution image (TIF 499 kb)

ESM 2 (PNG 2939 kb)

High resolution image (TIF 739 kb)

ESM 3 (PNG 1950 kb)

High resolution image (TIF 180 kb)

## Data Availability

The datasets generated and/or analysed during the current study are not publicly available as they require in-house reconstruction software but could be made available from the corresponding author on reasonable request.
