# Supplementary figures and images for: Imaging coronary plaques using 3D motion-compensated [18F]NaF PET/MR
Source: Eur J Nucl Med Mol Imaging. 2021 Jan 21;48(8):2455–65. doi: 10.1007/s00259-020-05180-4 (PMC8241750; doi:10.1007/s00259-020-05180-4)

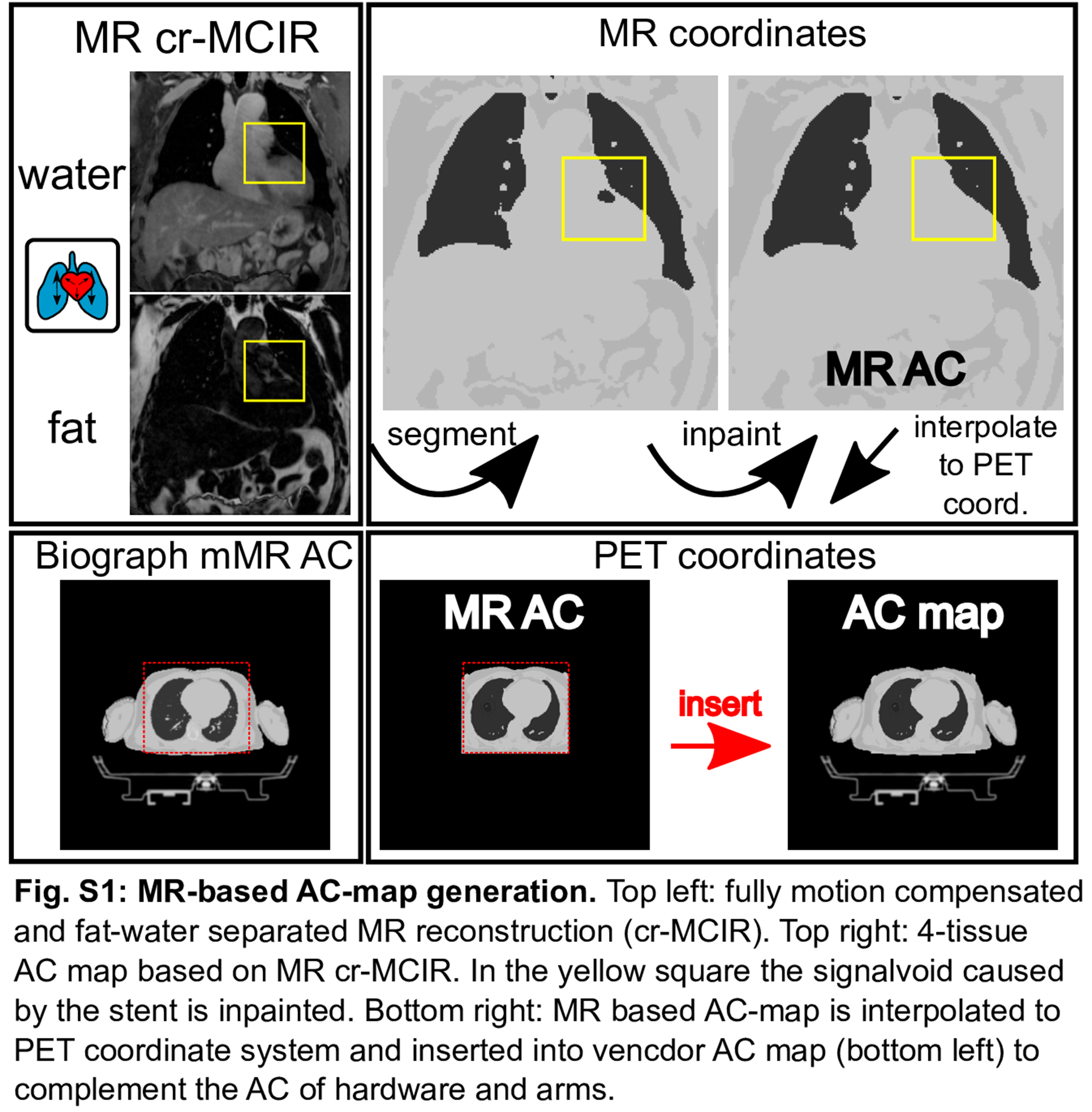

Supplement: Supplementary file 1 — (PNG 3881 kb) [file 259_2020_5180_Fig6_ESM.png]

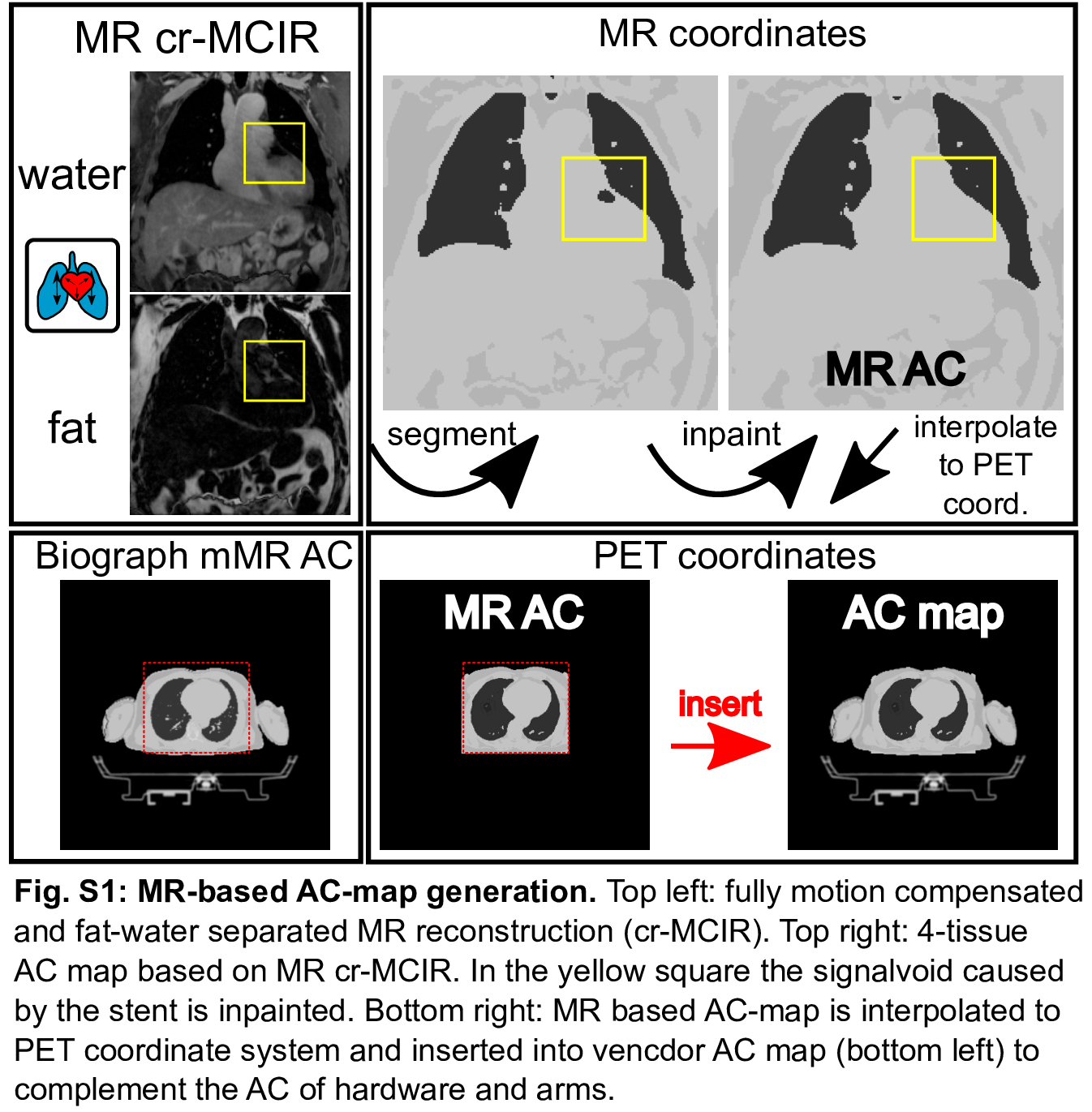

Supplement: Supplementary file 2 — High resolution image (TIF 499 kb) [file 259_2020_5180_MOESM1_ESM.tif]

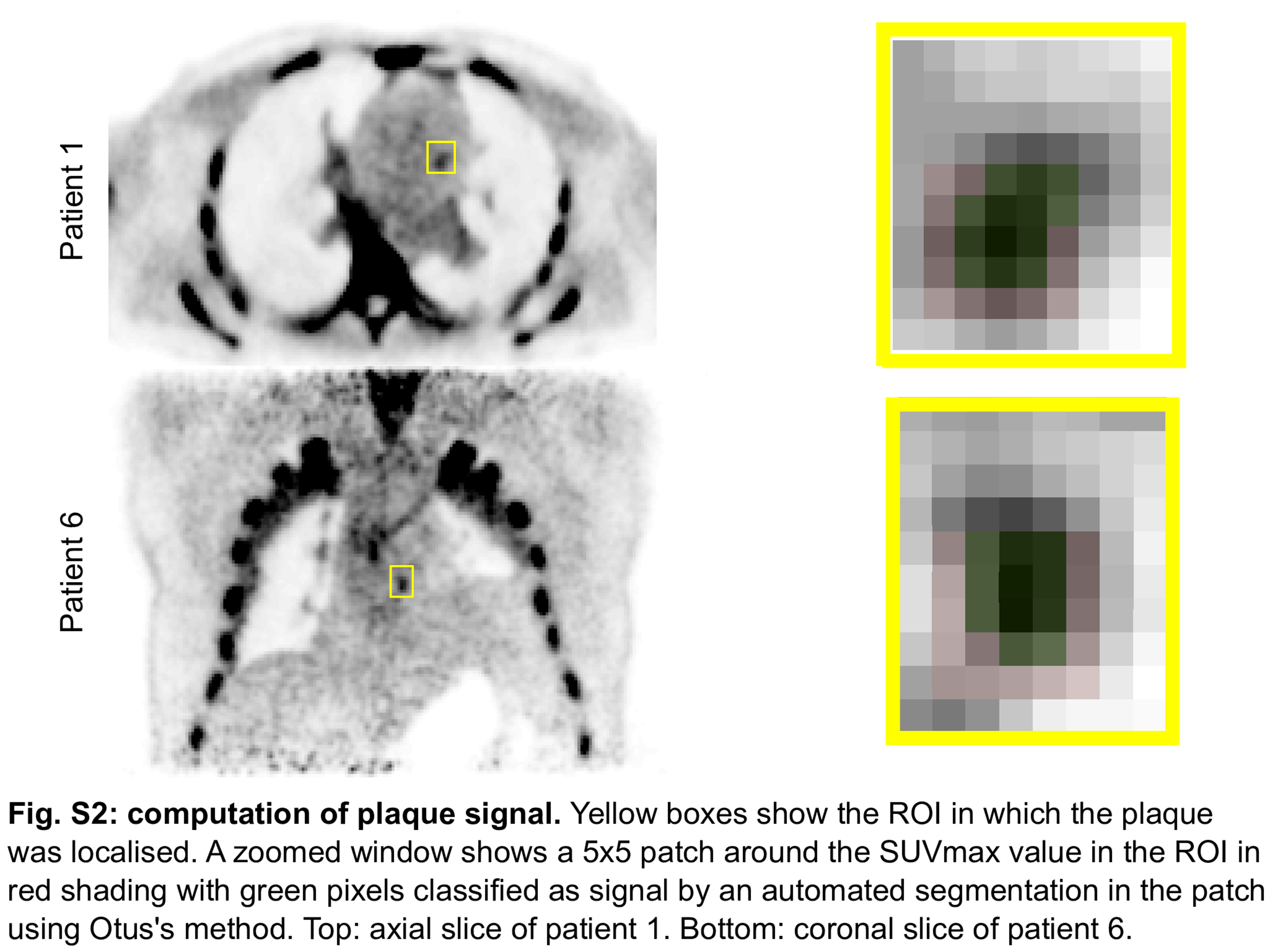

Supplement: Supplementary file 3 — (PNG 2939 kb) [file 259_2020_5180_Fig7_ESM.png]

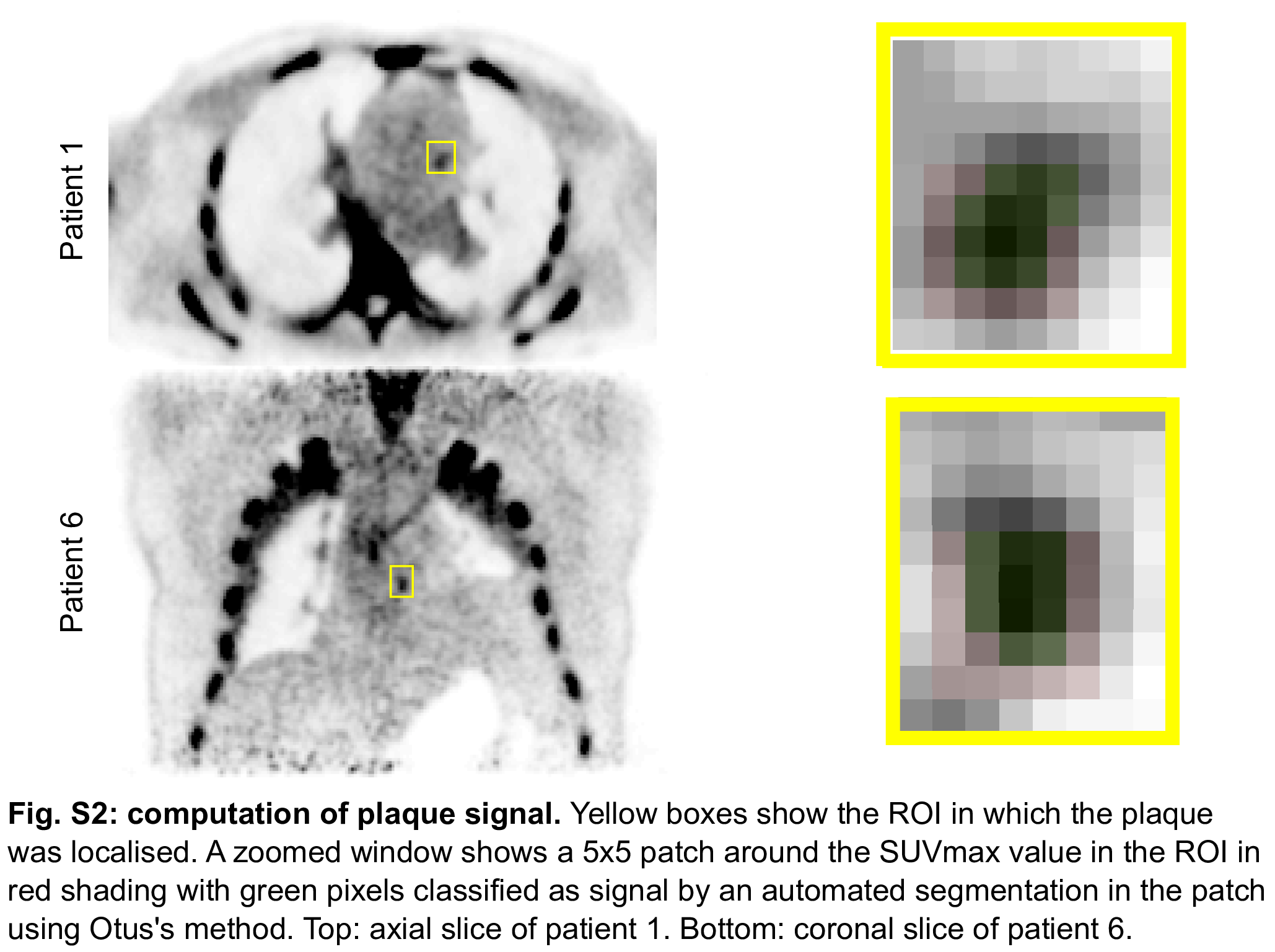

Supplement: Supplementary file 4 — High resolution image (TIF 739 kb) [file 259_2020_5180_MOESM2_ESM.tif]

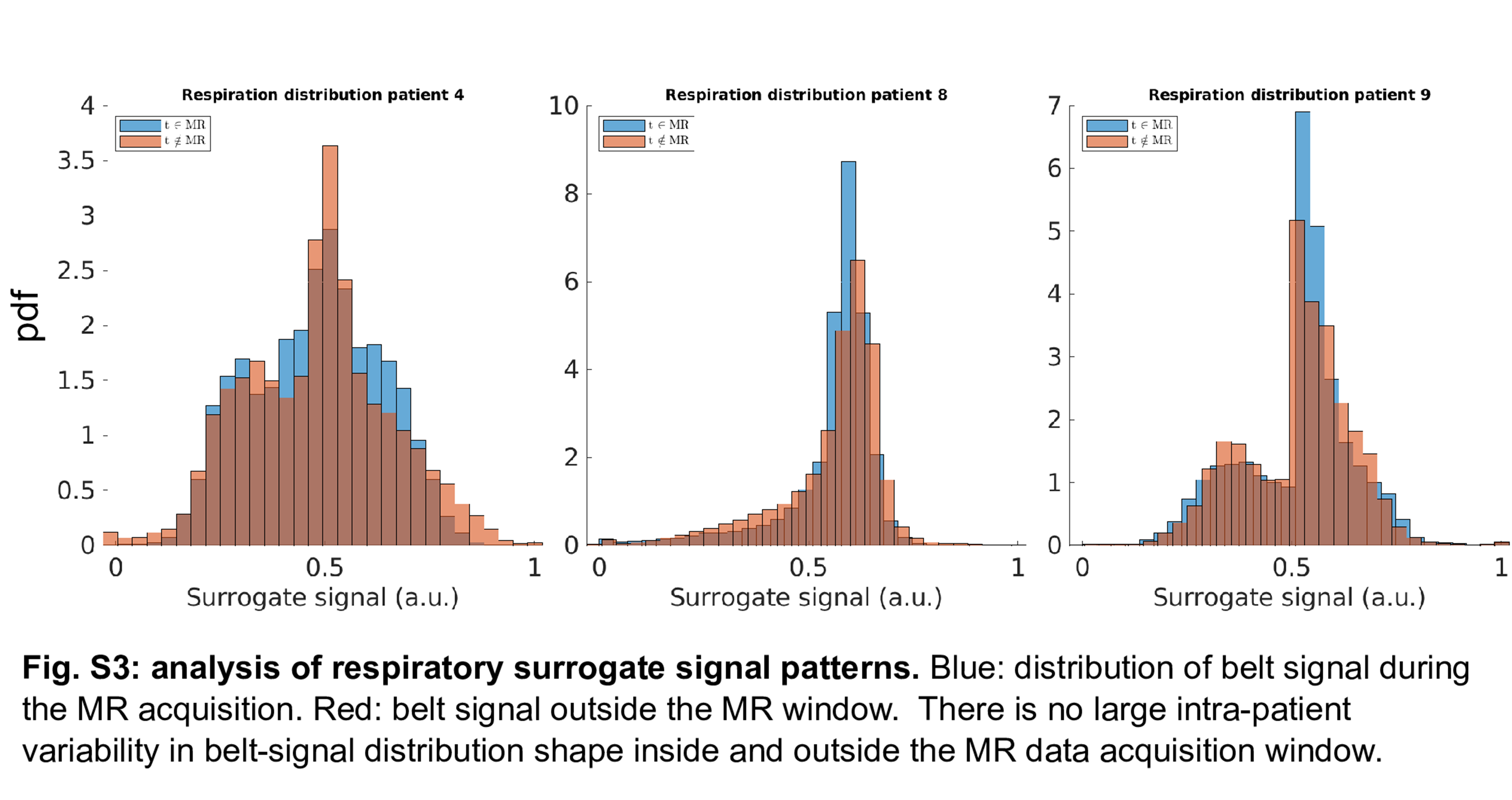

Supplement: Supplementary file 5 — (PNG 1950 kb) [file 259_2020_5180_Fig8_ESM.png]

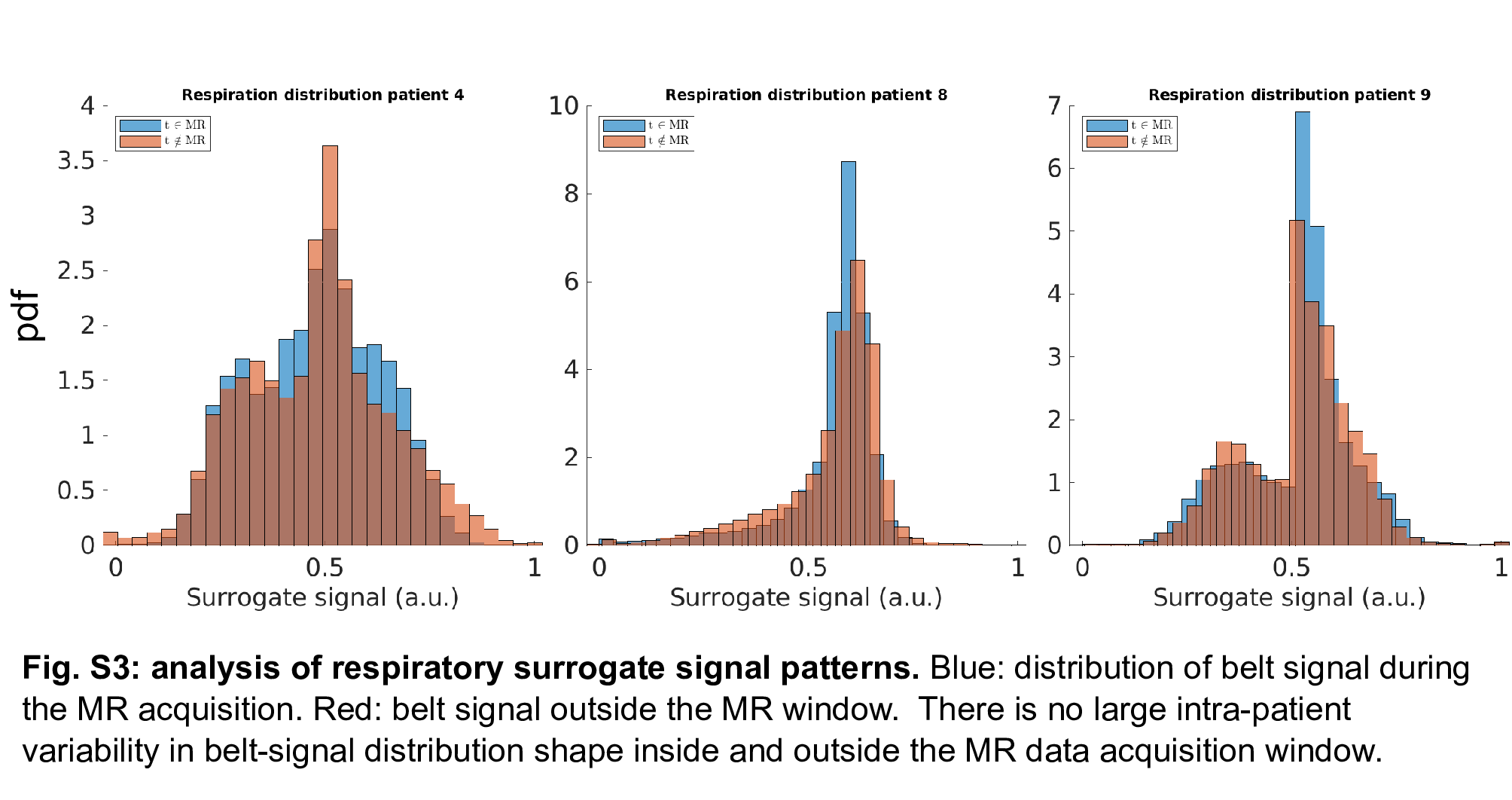

Supplement: Supplementary file 6 — High resolution image (TIF 180 kb) [file 259_2020_5180_MOESM3_ESM.tif]
